# Meeting Report: National Workshops for the Communication of Air Pollution and Health Information: Summary of Four Workshops in Different Regions of Europe

**DOI:** 10.1289/ehp.8524

**Published:** 2006-03-15

**Authors:** Eric Gordon Sanderson, Nina Fudge, Annike Irene Totlandsdal, Ingrid Hovelynck, Herbert Korbee, Edith Rameckers, Bert Brunekreef, Leendert van Bree

**Affiliations:** 1 Institute for Risk Assessment Sciences, Universiteit Utrecht, Utrecht, the Netherlands; 2 Netherlands Environmental Assessment Agency, Bilthoven, the Netherlands; 3 Korbee & Hovelynck BV, De Bilt, the Netherlands; 4 European Federation of Allergy and Airways Diseases Patients’ Association, Brussels, Belgium

**Keywords:** air pollution and health, communication, stakeholders, thematic network

## Abstract

AIRNET was a thematic network project (2002–2004) initiated to stimulate the interaction between researchers in air pollution and health in Europe. As part of AIRNET’s communication strategy, a standardized workshop model was developed to organize national meetings on air pollution and health (AIRNET network days). Emphasis was given to tailor the national workshop information and related activities to the specific needs of a wider range of stakeholders (e.g., policy makers, nongovernmental organizations, industry representatives). In this report we present an overview of the results of four workshops held in western, northern, central/eastern, and southern regions of Europe in 2004. Overall, workshop experiences indicated that by actively involving participants in the planning of each meeting, AIRNET helped create an event that addressed participants’ needs and interests. A wide range of communication formats used to discuss air pollution and health also helped stimulate active interaction among participants. Overall, the national workshops held by AIRNET offered a way to improve communication among the different stakeholders. Because a broad stakeholder involvement in decision making can positively affect the development of widely supported policies, such meetings should be continued for Europe and elsewhere.

A well-established body of evidence now shows that increasing levels of air pollution are linked with more illness, higher use of health services, and earlier death among the exposed population groups (Brunekreef and Holgate 2002). Recently, five disciplinary reports by AIRNET (Thematic Network on Air Pollution and Health) have addressed the evidence in the European Union (EU) from a variety of scientific perspectives, including epidemiology, toxicology, exposure assessment, health impact assessment, and the science–policy interface (AIRNET 2005a, 2005b, 2005c, 2005d, 2005e). Overall, these reports indicate that European research has significantly contributed to the better understanding of air pollution health effects.

AIRNET was a thematic network project (2002–2004) initiated to stimulate the interaction between air pollution and health researchers in Europe (AIRNET 2002). AIRNET collected, interpreted, and disseminated information from individual (EU-funded) projects to strengthen the science–policy interface and to draw policy-relevant recommendations. The objective of this stakeholder network was to create a widely supported basis for public health policy related to improving air quality in Europe—for instance, the communication of scientific findings for policy use and the identification of important gaps in the research. Overall, 23 project partners were initially brought into AIRNET, representing the scientific community and a variety of other stakeholders with an interest in air pollution and health.

Several reports stress the importance of stakeholder involvement in understanding the science at all stages of the decision-making process (Beierle 2002; Maynard et al. 2003; Tamburlini and Ebi 2002). Realizing the need for more stakeholder input, AIRNET strived to increase the number and diversity of participating stakeholders with varied interests deriving from a local, national, or regional perspective. To make the wealth of gathered and interpreted information available to a broader spectrum of stakeholders, two things were considered paramount: first, a fine-tuning of the information required to meet the needs of different stakeholders; and second, a well-focused effort undertaken to actively involve more stakeholders, including those who previously might not have had any contact with AIRNET.

Therefore, a major goal of the activities of AIRNET in its final year was to help bridge the gap between scientists, policy makers, and other relevant stakeholders. To this end, the communication strategy focused on the concept of national workshops (AIRNET network days). The workshop model gave the participants an opportunity to influence the planning of the meeting in line with their interests and needs. Ideally, such an approach should produce an atmosphere where stakeholders can comfortably create, broaden, and intensify their own personal network and can share their knowledge and questions. In this report we present the findings from four workshops organized to communicate and discuss air pollution and health issues specific to western, northern, central/eastern, and southern regions of Europe.

## Methods

Four countries (the Netherlands, Sweden, Hungary, and Spain) representing different European regions (western, northern, central/ eastern and southern) were selected to address region-specific air pollution and health issues in a standardized workshop format developed by AIRNET’s communication firm (Korbee & Hovelynck BV). Although the underlying approach used to organize the national workshops is a traditional management strategy, it is a little-used strategy in many scientific areas, especially for air pollution and health.

**Figure 1:**
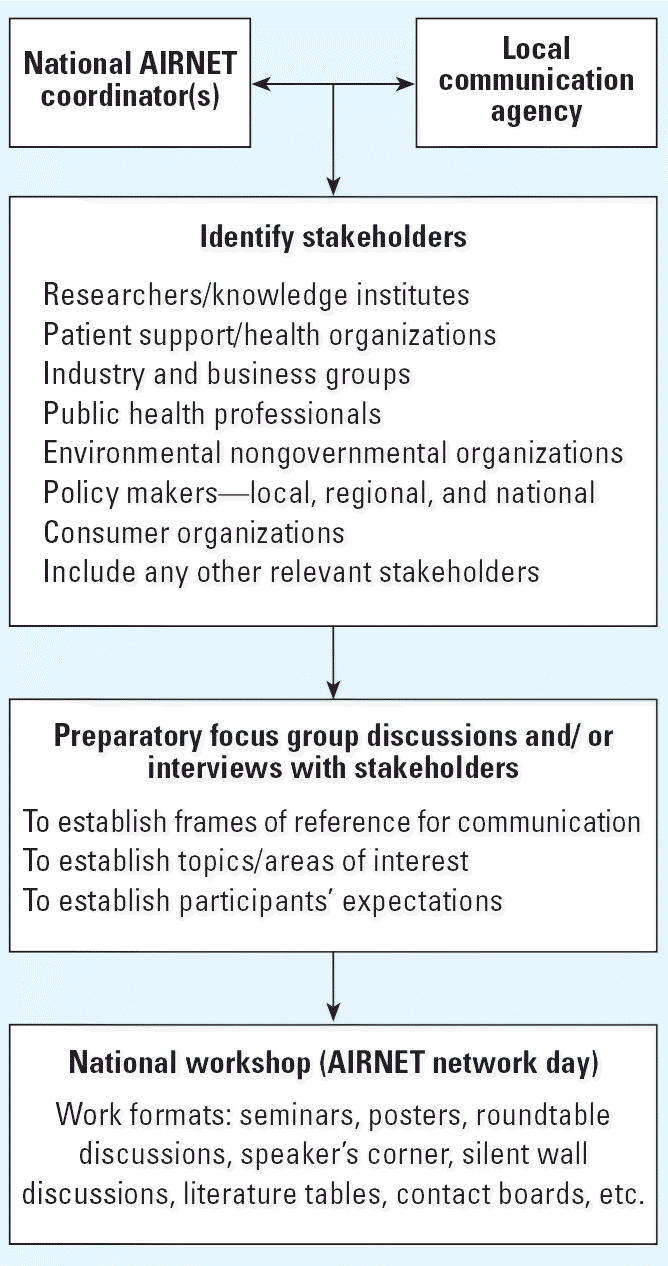
General schematic of the national workshop model.

As illustrated in Figure 1, the first step was for a national AIRNET coordinator (i.e., a scientist or a representative from a government agency) to select a local communication agency that could perform a stakeholder analysis to identify relevant target groups according to their interests in air pollution and health. The communication agency chosen was either a commercial public relations firm or a professional conference management firm with suitable experience in the field of public relations. Once the stakeholder list was compiled and preliminary invitations to the workshop were sent out, focus group discussions or interviews were held with representative stakeholders. The local communication agency organized these sessions and aimed to have stakeholder input from each stakeholder group identified in the stakeholder analysis.

The goal of the focus group discussions and interviews before the workshop was to understand what the stakeholders needed, how they could contribute to the meeting, and what the preferred means were for communicating and exchanging knowledge and opinions. There was also an opportunity to widen participation by asking stakeholders for the names of other interested parties who may have been missed in the initial stakeholder analysis. As the national workshops were held at different times throughout the year, we were able to build on the experiences and results of previous workshops to help develop subsequent events.

All the authors have been involved in the planning and participation of one or more of the workshops. For the overall descriptions, discussion, and evaluation of the workshops, the authors draw on their experiences and observations as well as any informal discussion with the participants.

## Results and Discussion

### Stakeholder participation

Participants at the workshops were classified into several stakeholder categories (Table 1): scientists (i.e., air quality, health) who perform research, policy makers (local, regional, national), industry representatives (i.e., automobile, oil, and gas), and nongovernmental organizations (NGOs) (e.g., patient rights, public health, and the environment). Additional stakeholders included participants who represented public transportation operators and clean fuel companies, and NGOs that advocated for public transport and cycling. Except at the Netherlands workshop, researchers were represented in the highest percentage. In general, policy makers were second, followed by NGOs and industry representatives—all of whom use research findings. Overall, AIRNET appears to have achieved a wide range of stakeholder participation at the national workshops between producers and users of research.

In general, the policy makers who attended the workshops represented national ministries dealing with the environment, meteorology and climatology, health care, and traffic. Other than those from a few of the municipalities where the meetings took place, few regional or municipal policy makers were present at the workshops. On the whole, the NGOs participating in the workshops represented a wide range of stakeholders, from consumer groups, environmental protection and management and environmental law, to environmental health advocacy for susceptible individuals (i.e., asthmatics and children). These NGO groups were typically functioning at the national interest level, sometimes under the umbrella of an international or pan-European parent organization.

Some stakeholder groups mentioned that international conferences (objectives, themes, content) are often biased toward researchers, making it less attractive for nonscientists to participate. Furthermore, some stakeholders groups (e.g., NGOs and local policy makers) find it difficult to attend international conferences because of budgetary and time constraints. This was demonstrated in the yearly AIRNET conferences, which were attended predominantly by scientists (Table 1). However, by offering local events where the attendee is involved in the design and setup, AIRNET has shown a way to increase the diversity of participation (see also Huntington et al. 2002). We are confident that workshops tailored to the participants’ interests increased the level of participation from all stakeholders, thereby demonstrating their potential usefulness as a medium through which to help develop consensus on research or policy.

### Workshop communication formats

The available work formats varied little from country to country, despite the fact that stakeholders were encouraged to indicate their preferred methods of communication. As indicated by the preworkshop focus group discussions and stakeholder interviews, the use of conventional presentation formats (seminar presentations, poster presentations) and roundtable discussions were favored. At three of the workshops, nonconventional activities (silent wall discussions, speaker’s corner, literature table, events calendar, contact board) were also used to stimulate stakeholder participation. Overall, every effort was made to ensure that the messages were relevant and easily understood to help stimulate stakeholder dialogue.

By using the proper meeting format, sharing knowledge can become more effective, widespread, and fine-tuned to meet different stakeholder needs (Huntington et al. 2002). However, the importance of selecting the most appropriate communication or work format for the intended audience is often overlooked by meeting organizers. In some instances, the use of conventional formats may be too passive to promote discussion. However, combined with more interactive methods, conventional formats can provide the information needed to fuel conversation. For example, silent wall discussions (reacting in writing to a statement on a blank poster) or a speaker’s corner presentation (analogous to a soapbox speech in London’s Hyde Park) can encourage people to become more active and allow alternative ways to participate.

Because of potential differences in air pollution and health issues and the communication styles among European regions, emphasis was given to tailoring information and work formats to the needs of the target groups. The workshops were also held in the national language, effectively removing potential barriers to communication caused by language. AIRNET’s experiences at each national workshop suggested that the selected work formats, room setups, chosen moderator, and rules for roundtable discussions were of prime importance in helping the stakeholder feel comfortable in contributing to the discussion. Overall, participants at the workshops reaffirmed the need to encourage successful two-way dialogue between stakeholders through both conventional and nonconventional communication methods.

### Major themes of stakeholder interest

Interviews and focus group discussions can provide better insight on stakeholder questions (Lion et al. 2002). Our focus group discussions and interviews produced a list of themes for workshop agendas that varied slightly by country (Table 2). The most prominent theme for all workshops was traffic-related air pollution and human health. Except for Spain, air quality standards were also of major interest. In addition, issues on asthma and allergy, as well as child/infant health, were a major focus for three of the five workshops. Except for Hungary, policy options aimed at air pollution and health were included in the program.

These initial themes were used to help promote attendance at each workshop but not necessarily to drive the direction of the discussion among participants. In the end, the Netherlands and Hungary roundtable discussions focused on the need to *a*) increase and improve public transportation, and *b*) encourage the public to take environmentally friendly steps to reduce the volume of traffic. Similarly, participants from the Swedish workshop indicated that health-orientated decision making would benefit from *a*) the development of traffic-related indicators of air quality, *b*) acute and chronic health effect studies for traffic, and *c*) integration of traffic and health policy with policies for air pollution reduction. For more detailed summaries of each workshop, see the AIRNET Web site (AIRNET 2002).

Although the workshop themes varied little among countries (Table 2), key messages emerging from each workshop were different in scope (Appendix 1). For instance, the effects of wood burning and spring dust were important topics in Sweden. In Hungary, many of the discussions focused on the health effects of ragweed exposure. Participants at the Netherlands workshop placed a greater focus on actions for the government and policy makers. In Spain, a dry climate resulting in dust production was an issue of importance among the attendees.

### European scope of the national workshops

A geographical spread (northern, western, central/eastern, and southern Europe) of national workshops allowed discussion of air pollution and health issues specific to each region. This aided in attracting policy makers and other stakeholders (e.g., environmental and health organizations) working at the local, regional, or national level (Table 1). A broad diversity of stakeholder perspectives helps improve decisions over the status quo by adding new information and ideas while ensuring adequate access to resources (Beierle 2002).

As a converging point for the national workshop activities on a European and regional level, answers to several questions posed during the parallel breakout sessions at the Third AIRNET conference are listed in Appendix 2. At this pan-European meeting, discussions relating to policy and decision-making priorities and the value of national or regional meetings reinforced AIRNET network activities (workgroup meetings, conferences, AIRNET network days). Overall, for all regions of Europe, improved communication between scientists, decision makers, and stakeholders was seen by the participants as highly desirable to increase the effectiveness of decision-making processes for environmental health improvement.

### Participant feedback

Participant feedback from the workshops was positive. On a scale of 1 to 10 (where 1 is bad and 10 is good), the overall ratings by participants in the Netherlands, Sweden, and Spain were 7.9, 7.2, and 8.1, respectively. (No rating was available for Hungary.) Most participants at each workshop felt that the objectives of the day, to exchange knowledge and strengthen personal networks, were well achieved. Moreover, participants were positive about the work formats used (specifically the roundtable discussions), despite not having worked in such formats before. Overall, most participants felt that it would be valuable to hold events of this type in the future, providing valuable feedback for the organizers.

The feedback sheets from the workshops contained numerous suggestions and ideas for the future, some of which are summarized below:

- Events should be longer and contain more scientific lectures and discussions.
- Circle of participants should be wider.
- Such events should be continued, where different sectors, interest groups, and stakeholders communicate with each other and with the public.
- Students dealing with health issues and protection of the environment should participate.

### Usefulness of national workshops

A dynamic science–stakeholder–policy interplay is needed to achieve successful air pollution abatement measures to decrease health risks. This interplay is important when developing sustainable policies that are transparent and sound and that carry the support of the policy makers, researchers, other stakeholders (industry and NGOs), and the general public. AIRNET attempted to incorporate the needs and views of all players involved during its 3-year existence. To help get this interplay running, the national workshops were used to improve communication between all players and to better understand each other’s needs.

Traditionally, expert workshops in Europe and internationally have been used to discuss the scientific and policy issues related to ambient air quality and human health [Bell et al. 2002; O’Neill et al. 2003; World Health Organization (WHO) 2000, 2001, 2003]. Agencies, and the WHO in particular, regularly perform expert reviews of air pollution and health information (WHO 2000, 2001, 2003). In comparison, relatively few national workshops are organized by involving the stakeholders in the planning of the meeting itself. Moreover, there is a scarcity of publications offering clear guidance and suggestions on how to organize and conduct multiparticipatory workshops for a heterogeneous group of individuals. The workshops organized by AIRNET included a wide range of stakeholders. Crucially, a representative subset of the invited stakeholders was involved in the development of the meeting itself.

By organizing events aimed at bringing a diverse audience together, AIRNET gave stakeholders a responsibility to create and broaden their own personal network, and to share their knowledge and questions. The national workshops were a good opportunity for stakeholders to try out different modes of communication. Overall, we believe that the workshops held by AIRNET were a step forward in stakeholder engagement in the field of air pollution and health and a clear response to discussions from previous meetings on the use of scientific knowledge in decision making (Ginsburg and Cowling 2003; Samet and Lee 2001).

## Concluding Remarks

How will such workshop activities continue? Who has the time and inclination to organize them? How is discussion translated into action?

Answers to the above questions are challenging, given AIRNET’s initial focus and the relatively short mandate given by the EU (3 years). Nonetheless, these issues were dominant at the final AIRNET conference, where attendees clearly wanted events that enhanced participant interaction (Appendix 2). However, since AIRNET officially ceased to exist in 2005, other supporters (EU, national governments, NGOs, industry, etc.) will need to take the initiative and time to organize, sponsor, and promote similar events. Doing so may help ensure that future actions are taken by maintaining a direct link with those who will be making the decisions. Although the issues are often big and the meeting times relatively short, events such as national workshops can be seen as a beginning or continuation of the existing dialogue and debate.

A limitation of the workshops (which were not initially part of AIRNET’s planned activities) is that they did not examine the steps needed to achieve subsequent actions resulting from the workshops. However, a legacy of these activities is the creation of a stakeholder network that will continue to interact (at some level) through other means. For example, many of the participants of the workshop in Spain decided that they would continue to communicate and work toward collectively feeding into policy debate in their country. Reducing environmental exposures may also require substantial financial investment, where broad support by a variety of stakeholders can be achieved through meaningful, relevant, and understandable communication. For a variety of reasons, this publication of the meetings summary is a step toward action rather than just keeping ideas merely at the level of discussion. This is important, if not critical, for comprehensive and sound management of any real or perceived risk to the human health (Jardine et al. 2003).

Well-planned and -moderated workshops can enhance communication and knowledge sharing among individuals who do not know each other well (or at all) and have different levels of understanding (Huntington et al. 2002; Kontic et al. 2006). Through activities such as the AIRNET national workshops, we believe that a substantial contribution to research planning or influencing policy can be achieved by ensuring that

- Stakeholders are familiar with the extent of the knowledge base (and its limitations or gaps) and how to gain access to this information.
- Stakeholders are able to use the information (available in a suitable format) for practical application in their own fields of specialty (Sanderson et al., in press).
- Stakeholders know whom to turn to with specific questions and will do so actively.
- Stakeholders know with whom they can share their acquired knowledge to maximize the impact of their efforts and help others in their pursuits.
- Stakeholders have a sufficient understanding of the subject matter under policy scrutiny to make a constructive and positive contribution to the decision-making process.

In conclusion, we feel that the national workshops were highly valuable in promoting participant interaction and improving communication among a wide range of stakeholders. Herein, active participation is key to enable a two-way flow of information. By bringing together the relevant stakeholders, well-planned workshops can empower a group of individuals who share a common interest or vision to participate collectively in the policy debate.

## Figures and Tables

**Table 1:** Overview of national workshop attendance by stakeholder category.

			Stakeholder category					
Workshop	Meeting length (hr)	No. of participants	Research (%)	Industry (%)	(%)	Environment (%)	Policy (%)	Transport and mobility (%)
Netherlands	4	52	23	12	10	8	42	6
Sweden	5.5	54	56	13	2	4	20	6
Hungary	5	40	43	3	18	15	10	13
Spain	6	39	20	5	35	20	10	0
3^rd^ Annual Conference	2.5 days	138	64	9	7	5	15	—

**Table 2:** Major themes of interest included in the national workshop programs.

Workshop	Traffic	Allergy and asthma	Children/infant health	Indoor air quality	Air quality standards	Other policy options
Netherlands	x				x	x
Sweden	x			x	x	x
Hungary	x	x	x	x	x	
Spain	x		x			x
3^rd^ Annual Conference	x	x	x	x	x	x

## Appendix 1. Examples of key messages resulting from the national workshops

### Netherlands (western Europe) network day:

- Development of an integrated air pollution and climate policy.
- Harmonization and validation of the models used to assess urban air quality.
- Investigation of methods to protect the environment, with open options for managing local hotspots.

### Sweden (northern Europe) network day:

- Need for action and research is most important for particulate matter from road and tire wear.
- Health effects of ozone have been somewhat forgotten—a need to refocus some attention.
- Maintainance of an air quality standard for the coarse fraction of particulate matter.

### Hungary (central/eastern Europe) network day:

- Air quality limit value system should be as dynamic as possible and monitored continuously.
- Means of transportation and urban planning should be developed in consideration of health issues.
- Allergy is the endemic of the 21st century, necessitating suitable preventative measures.

### Spain (southern Europe) network day:

- Need for a professional organization to oversee monitoring station criteria for the EU.
- Promotion for the reduction of air pollution (i.e., urban planning, technology, public information).
- A shift from epidemiological surveillance to more vigilance toward lifestyle risk factors.

Network day summaries in English are available on the AIRNET Web site (http://airnet.iras.uu.nl/).

## Appendix 2. Summary of the Third AIRNET Conference regional breakout sessions

### Within our region, what air pollution and health problems and abatement needs do we have in common?

- Northern Europe: soil dust, noise, and traffic-related emissions.
- Western Europe: primarily particulate matter, although nitrogen dioxide is still an issue.
- Central/eastern Europe: air quality affected by local heating and traffic.
- Southern Europe: traffic-related emissions, desert dust, and secondary air pollutants.

### What have we learned from this conference that we can take home/apply to our region?

- Northern Europe: promotion of bicycle use to decrease traffic-related air pollution.
- Western Europe: lack of clear communication among scientists, policy makers, and stakeholders.
- Central/eastern Europe: improvement in the public understanding of environmental health issues.
- Southern Europe: good public transportation is needed to decrease traffic in cities.

### What are the research priorities for our region?

- Northern Europe: confirmation of the improvement in air quality as a result of implemented policy.
- Western Europe: carrying out long-term health studies of low-concentration air pollutants.
- Central/eastern Europe: impact of changing air pollution on human health, especially in children.
- Southern Europe: research targeted on defining source and composition of air pollution.

Session summaries in English are available on the AIRNET Web site (http://airnet.iras.uu.nl/).
